# Benzene Exposure From Selected Work Tasks on Offshore Petroleum Installations on the Norwegian Continental Shelf, 2002–2018

**DOI:** 10.1093/annweh/wxac067

**Published:** 2022-10-21

**Authors:** Hilde Ridderseth, Dagrun Slettebø Daltveit, Bjørg Eli Hollund, Jorunn Kirkeleit, Hans Kromhout, Kirsti Krüger, Kari Aasbø, Magne Bråtveit

**Affiliations:** University of Bergen, 5020 Bergen, Norway; University of Bergen, 5020 Bergen, Norway; University of Bergen, 5020 Bergen, Norway; University of Bergen, 5020 Bergen, Norway; Institute for Risk Assessment Sciences, Utrecht University, 3584 CS Utrecht, The Netherlands; Equinor ASA, 4035 Stavanger, Norway; Equinor ASA, 4035 Stavanger, Norway; University of Bergen, 5020 Bergen, Norway

**Keywords:** cancer, determinants, full-shift exposure, job group, occupational benzene exposure, offshore installation, petroleum industry, short-term exposure, time trend, work task

## Abstract

**Objectives:**

Work on offshore petroleum installations may cause exposure to benzene. Benzene is a carcinogenic agent, and exposure among workers should be as low as reasonably practicable. We aimed to assess short-term (less than 60 min) benzene exposure from the most frequent work tasks on offshore installations on the Norwegian continental shelf and identify determinants of exposure. In addition, we aimed to assess the time trend in task-based benzene measurements from 2002 to 2018.

**Methods:**

The study included 763 task-based measurements with a sampling duration of less than 60 min, collected on 28 offshore installations from 2002 to 2018. The measurements were categorized into 10 different tasks. Multilevel mixed-effect Tobit regression models were developed for two tasks: sampling and disassembling/assembling equipment. Benzene source, season, indoors or outdoors, design of process area, year of production start, sampling method, and work operation were considered as potential determinants for benzene exposure in the models.

**Results:**

The overall geometric mean (GM) benzene exposure was 0.02 ppm (95% confidence intervals 95%(CI: 0.01–0.04). The pipeline inspection gauge (PIG) operation task was associated with the highest exposure, with a GM of 0.33 ppm, followed by work on flotation cells, disassembling/assembling, and sampling, with GMs of 0.16, 0.04, and 0.01 ppm, respectively. Significant determinants for the disassembling/assembling task were work operation (changing or recertifying valves, changing or cleaning filters, and breaking pipes) and benzene source. For sampling, the benzene source was a significant determinant. Overall, the task-based benzene exposure declined annually by 10.2% (CI 95%: −17.4 to −2.4%) from 2002 to 2018.

**Conclusions:**

The PIG operation task was associated with the highest exposure out of the ten tasks, followed by work on flotation cells and when performing disassembling/assembling of equipment. The exposure was associated with the type of benzene source that was worked on. Despite the decline in task-based exposure in 2002–2018, technical measures should still be considered in order to reduce the exposure.

**What is important about this paper**

This study provides updated knowledge on task-based benzene exposure at offshore petroleum installations (2002–2018). Overall GM exposure was 0.02 ppm benzene, with the highest exposures associated with the pipeline inspection gauge operation task (GM 0.33 ppm). These data can be used to identify where controls should be implemented to reduce exposure.

## Introduction

Workers on offshore petroleum installations are at risk of exposure to benzene when performing tasks in the process area. Benzene is classified as a leukemogenic agent (IARC, 2018) and is a natural component in the petroleum streams when producing oil and gas.

Previous studies have reported full-shift exposure to benzene among offshore petroleum workers to have a geometric mean (GM) ranging from 0.004 ppm to 0.036 mg/m^3^ [0.011 ppm], (Verma *et al.*, 2000; Kirkeleit *et al.*, 2006; Bråtveit *et al.*, 2007; Ridderseth *et al.*, 2022). This is below the present occupational exposure limit value (OELV) in the EU of 0.2 ppm (1 ppm in a transitional period until April 2024 and 0.5 ppm until April 2026) for an eight-hour working day (European Union, 2022). However, epidemiological studies among upstream petroleum workers have reported an excessive risk of leukaemia at exposure levels below this OELV (Glass *et al.*, 2003; Stenehjem *et al.*, 2015). The American Conference of Governmental Industrial Hygienists (ACGIH) has created a Notice of Intended Changes List (NIC) for 2022 which proposes OELV at 0.02 ppm (ACGIH, 2022). According to the Norwegian *Regulations concerning the Performance of Work* (Ministry of Labour and Social Inclusion, 2013), the exposure to carcinogens should be ‘as low as reasonably practicable’ (ALARP) when it is not possible to phase out or retain the chemical in a closed system (Ministry of Labour and Social Inclusion, 2013). Although full-shift benzene exposure might be low compared to the present OELV in Europe, measures should thus be considered and implemented to reduce exposure even further.

The processing of the petroleum stream on offshore installations in principle takes place in a closed system, but the system must be opened during maintenance, repair, and collection of samples. Tasks associated with benzene exposure include sampling from the petroleum streams and disassembling/assembling process equipment, including breaking pipes, and equipment. There is a general lack of published task-based benzene measurements from the offshore petroleum industry for the Norwegian Continental Shelf (NCS) and globally. Nonetheless, short-term (sampling duration: 3–76 min) measurements from 2005 indicated relatively high exposure to benzene during specific tasks such as work on the system separating water from the oil (GM = 0.77 ppm), cleaning pipeline pigs (0.26 ppm) and opening process equipment (0.17 ppm) (Bråtveit *et al.*, 2007). However, the exposure measurements in Bråtveit *et al.* (2007) were collected on only one offshore installation, and the actual determinants of exposure such as benzene source, season, indoor or outdoor work, design of process area, and year of production start for these tasks were not examined.

The present study aims to assess short-term benzene exposure for typical work tasks in the offshore petroleum industry on the NCS, identify determinants of task-based exposure, and assess long-term temporal trends in task-based measurements collected from multiple offshore installations over a period of almost two decades (2002–2018).

## Methods

### Dataset

**Figure 1.**
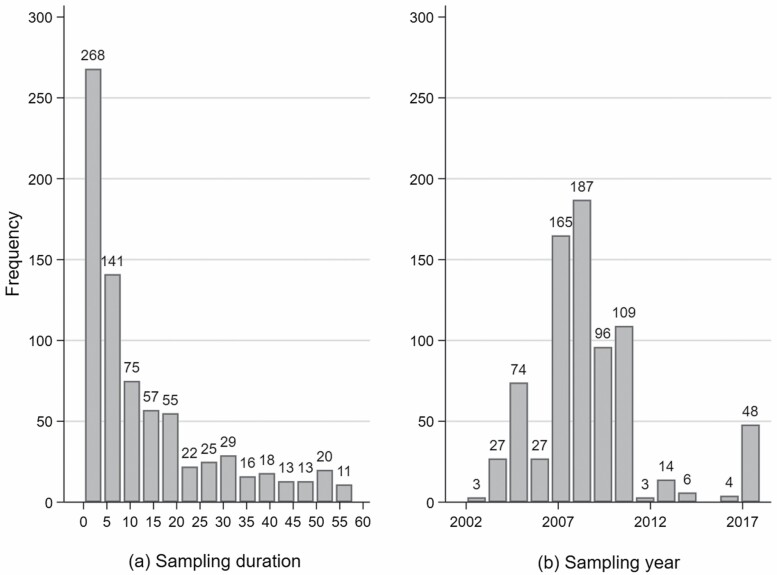
(a) The frequency of sampling duration (minutes) and (b) sampling year (2002–2018) for the benzene measurements included in the dataset (28 offshore installations on the Norwegian continental shelf).

The dataset comprised personal benzene measurements from workers employed at upstream offshore installations on the NCS. The dataset included 763 personal benzene measurements from 86 reports for surveys performed at 28 offshore installations from 2002 to 2018, mainly from one oil company. The primary job groups with measurements were process operators, laboratory technicians, and mechanics. In this study, only measurements with a sampling duration below 60 minutes (min) were included, as these samples were most likely to have been collected during the performance of specific tasks. The frequency of sampling duration and sampling year for the total dataset are presented in Fig. 1. Included in the dataset were also 26 measurements from 2005 with sampling duration below 60 min reported by Bråtveit *et al.* (2007). These measurements represent the following tasks: pipeline inspection gauge (PIG) operation, work on flotation cells, breaking pipes, and collecting samples from the petroleum stream with 5, 10, 4, and 7 measurements, respectively.

### Description of the benzene sources

Crude oil, condensate, and gas from the NCS are extracted from the reservoir under the seabed. These components, in addition to water and solids, enter the offshore installation’s well section and are transported to the processing area for separation into different petroleum streams. The petroleum streams contain benzene and other substances, depending on the geological formation of the reservoir and the maturity of the oil field. The petroleum industry produces large amounts of wastewater (produced water) that must be purified, that is oil droplets must be removed (DNVGL, 2015). The benzene content of the produced water varies throughout the stages of the separation process. According to the company’s assays, the benzene content of the crude oil blend in our study ranged from 0.01 to 0.90 weight percent (wt%). Condensates are present in natural gas, crude oil, and as a product of gas drying. The benzene content of five condensates from the NCS was reported to vary between 0.42 and 1.98 wt% (Gjesteland *et al.*, 2019), while the content of benzene in produced water and natural gas is lower (<0.1 wt%) (Norwegian Oil and Gas Association, 2014). Benzene is also present during the gas drying process, where glycol is used as a dehydrator. Glycol absorbs benzene, among other components, and might contain 0.1–1 wt% benzene after the gas drying process. Used glycol is called ‘wet glycol’ (Norwegian Oil and Gas Association, 2014).

### Tasks with potential for benzene exposure

The measurements were categorized into 10 different tasks (Table 1). Some of the tasks also consist of subgroups referred to as *work operations*.

**Table 1.** Description of tasks and work operations associated with exposure to benzene.

Task	Work operation	*N*	Sampling duration, median minutes	Description	Job group (*n*)
			(range in minutes)		
Total		763	7 (0.5–58)		Laboratory technicians (305) Process operators (293) Mechanics (116) Others(47) Industrial cleaners (2)
Sampling		355	5 (0.5–56)	Collecting samples from the petroleum streams.	Laboratory technicians (255) Process operators (85) Others (12) Mechanics (3)
Disassembling/assembling	All four work operations listed below	154	20 (1–58)	Dissembling/assembling equipment, including breaking pipes, and changing or cleaning filters.	Mechanics (91) Process operators (58) Others (5)
	Recertification or changing valves	25	30 (3–58)		
	Changing or cleaning filters	63	12 (1–58)	The process system contains different types of filters that are in contact with varying streams of products that contain benzene.	
	Breaking of pipes	35	18 (6–45)		
	Other disassembling/assembling tasks	31	18 (2–55)		
Laboratory work		71	8 (0.5–55)	Analysing samples from the process flow, such as control of oil-in-water in the produced water.	Laboratory technicians (42) Others (16) Process operators (13)
PIG operation		28	15 (4–46)	Receiving or transmission of PIG	Process operators (28)
Control of sand trap		33	4 (1–11)	Flushing and collecting samples	Process operators (32) Laboratory technicians (1)
Skimming		23	7 (1–30)	Flushing and collecting samples	Process operators (21) Laboratory technicians (2)
Working on hydrocyclones		19	4 (1–53)	Opening, cleaning, and sampling.	Process operators (9) Mechanics (8) Laboratory technicians (2)
Working on flotation cells		16	6 (2–15)	Inspection and maintenance of the flotation cells	Process operators (14) Laboratory technicians (1) Mechanics (1)
Bleeding off pressure		15	8 (1–45)	A small amount of gas will be released before opening equipment or breaking pipes.	Process operators (11) Mechanics (1) Others (3)
Other tasks^*a*^		49	17 (1–53)	Including a wide range of tasks	Process operators (22) Others (13) Mechanics (12) Laboratory technicians (2)

*N*: the number of measurements, *n*: the number of measurements in the job group for each task.

^*a*^ *Other tasks* includes: check of prover ball, manual cleaning, drainage, refilling, jetting, unknown tasks, and safety guard (during tank work).

The *sampling from the petroleum streams* task is carried out several times a day by laboratory technicians or process operators (Bråtveit *et al.*, 2007). In the process area, crude oil samples are either collected via an automated short-cut loop or manually from taps into small bottles. Collection of one sample typically takes 1–5 min. The total work operation for collection of several samples usually lasts 30–40 min and is generally conducted twice a day.

**Figure 2.**
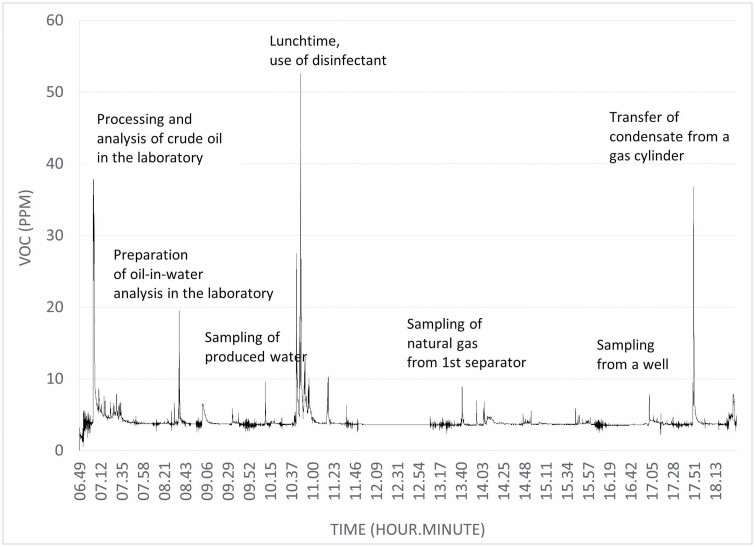
A laboratory technician’s exposure to volatile organic compounds VOC (ppm, as isobutylene equivalents) during several work tasks performed during a typical work shift.

The *laboratory work* task includes indoor work to perform quality tests such as analysis of water content in crude oil, specific weight of crude oil and condensate, and oil content in produced water (Bråtveit *et al.*, 2007). The work is usually performed in air extraction cabinets. Fig. 2 shows the air concentration of volatile organic compounds monitored by a direct reading device carried by a laboratory technician during a full shift. Benzene is presumably part of the peak exposures in Fig. 2.

The *bleed off the pressure* task is performed on part of the process system and must be performed before disassembling the process equipment for maintenance. A small amount of gas is released, leading to potential exposure to benzene. Transmitters measuring pressure and volume in separators or tanks must also occasionally be bled off before recalibration and testing. The duration of gas release typically lasts from a few seconds to a couple of minutes.

The *disassembling/assembling* task includes work operations performed mainly by mechanics, comprising breaking pipes or isolating a segment of the petroleum system by placing or removing blind flanges (spades). Maintenance and repair of pumps and compressors are also classified as ‘disassembling/assembling’. Pressure relief valves are brought into the workshop for control. The *changing and cleaning filters* work operation is mainly performed by process operators in the process area and is included in disassembling/assembling.

A broad range of water treatment systems is used to rinse the produced water on offshore installations (DNVGL, 2015). The present study includes measurements taken during performance of tasks such as *work on flotation cells*, *hydrocyclones*, and *skimming* (Table 1). In the *flotation cells*, the oil residue is skimmed off. Exposure might occur if the system does not function as intended; in such case, inspection by opening the inspection door is required to assess whether it needs to use a swab to push the oil phase over the separation edge (Bråtveit *et al.*, 2007). To control the oil content in the water from the flotation cells, water samples are taken from an open tap. The *work on hydrocyclones* task can lead to exposure when hydrocyclones are opened for cleaning. The frequency at which the system is opened depends on the oil content of the produced water and the amount of scale on the interior surface of the system. The hydrocyclones are typically cleaned every third month, but less frequently for installations with automatic cleaning systems. Samples from produced water are regularly taken to control the quality of the rinsed water. The *work on the skimming system* task might lead to exposure during flushing and collection of samples.

The *control of the sand trap* task includes work operations such as cleaning and emptying the sand trap. Process operators might be exposed to vapour from hot-produced water containing benzene. A sand trap that removes sand particles from the petroleum stream is usually located before the first separator tank.

The *PIG operation* task comprises inserting and removing the PIG. The PIG is a device to remove scale or inspect pipelines. Before inserting a PIG, the operators depressurize the PIG launching station and open the station door. The operation lasts around 15 min. Similarly, the system is depressurized and opened before the operators remove a PIG from the receiver station, either by hand or with mechanical assistance. The task duration for removing a PIG is around 30 min (Bråtveit *et al.*, 2007). The frequency of inserting and receiving a PIG varies from installation to installation.

Tasks with less than 10 measurements were grouped as *other tasks* (e.g. fuelling diesel and jet fuel, handling small quantities of oil-contaminated waste, checking prover ball, manual cleaning, jetting, and tank cleaning).

### Preparation of data

Benzene source was categorized into six groups based on the nature of the source: crude oil, condensate, produced water, wet glycol, mixed benzene sources, and other benzene sources. The design of the installations was classified as open, partially restricted, and restricted. Open means that the process area has no walls and is naturally ventilated. Partially restricted comprises installations with walls in parts of the process area, while restricted is closed with walls and limited affect from natural ventilation (Lloyd, 2016). The year of production started was divided into three decades: 1979–1989, 1990–1999, and 2000–2005. The seasons were divided into winter (October–March) and summer (April–September). The site categorizes whether the work was performed indoors, outdoors, or inside a tank. Indoor work usually takes place in a mechanical workshop or in a test station for pressure safety valves, while outdoor work takes place in the process area. The sampling methods were classified as passive (3M dosimeter or automated thermal desorption tubes [ATD]) or active (ATD or charcoal tubes with pumps).

Around 23% of the measurements were below the limit of detection (LOD) which range from 0.0001 to 0.18 ppm. Exposure measurements reported as mg m^−3^ in the reports were converted to ppm using the formula: ((mg m^−3^ × 24.06)/78.11 mol).

### Statistical analyses

The data were log-transformed before analysis due to right-skewed distribution. To account for different LODs and measurements with results below LOD, tobit regression was used (Lubin *et al.*, 2004; Lotz *et al.*, 2013). Multilevel mixed-effect tobit regression was used to estimate GM, GM ratios, and 95% confidence intervals. The log-transformed concentration of benzene was used as a dependent variable and installation as a random effect. ‘Worker ID’ was recorded for only 32 workers in 123 of 763 measurements, of whom 23 workers out of the 32 workers had measurements from more than 1 day. We thus considered not having enough repeated measurements to estimate within-worker and within-day variance.

The unadjusted analyses were stratified by tasks, benzene source, season, indoor or outdoor work, design of process area, year of production start, and sampling method. To identify determinants of exposure, we first developed a random-effect model with between- and within-installation variability. Then two multilevel mixed-effect tobit regression models for benzene exposure were developed to identify determinants for the two most frequently measured tasks: disassembling/assembling and sampling. All variables from Table 2 and, in addition, the sampling duration were considered for inclusion in the models. However, the year of production start was highly correlated with the installation’s design, whereby newer installations were more open and older installations were more restricted. The year of production start was therefore excluded from the model. The exposure model for disassembling/assembling included three common work operations as fixed effects: recertification or changing valves, changing or cleaning filters, and breaking pipes. Backward elimination was performed, and fixed effects with a significance level below 0.20 were kept in the models. The total variance was estimated by adding the variance between installations (_bp_S^2^) and within installations (_ww_S^2^). The percentage reductions of exposure variability between and within installations due to the inclusion of fixed factors were estimated. One supplementary model, including all the variables in (Supplementary Table S2), in addition to sampling duration and excluding the year of production start, was developed. These models are intended for future development in the industry when more task-based measurements are made available.

**Table 2.** Unadjusted estimates of GM benzene exposure in ppm, GM (95% CI), and GM ratio using the mixed-effect model. In addition, the minimum and maximum exposure in each group and arithmetic mean in ppm.

Characteristic	*N*	% <LOD	Min–max	AM (95% CI)	GM (95% CI)	GM ratio (95% CI)
			ppm			
Overall exposure	763	23	<LOD–22.2	0.35 (0.26–0.43)	0.02 (0.01–0.04)	
**Work operation**						
Sampling	355	29	<LOD–11.7	0.32 (0.19–0.49)	0.01 (0.01–0.03)	Reference
Disassembling/assembling	154	8	<LOD–22.2	0.50 (0.20–0.80)	0.04 (0.02–0.10)	3.56 (2.03–6.26)
Laboratory work	71	49	<LOD–1.50	0.07 (0.02–0.13)	0.01 (0.002–0.01)	0.38 (0.17–0.86)
Control of sand trap	33	55	<LOD–0.40	0.08 (0.38–0.12)	0.01 (0.002–0.02)	0.51 (0.17–1.54)
PIG operation	28	0	0.03–10.0	0.59 (0.24-0.95)	0.33 (0.09–1.14)	26.72 (8.56–83.5)
Skimming	23	13	<LOD–0.71	0.13 (0.05–0.22)	0.03 (0.01–0.12)	2.48 (0.76–8.29)
Work on hydrocyclones	19	21	<LOD–0.62	0.14 (0.52–0.23)	0.03 (0.01–0.11)	2.06 (0.55-7.69)
Work on flotation cells	16	6	<LOD–2.33	0.73 (0.32–1.13)	0.16 (0.03–0.82)	13.05 (2.76–61.8)
Pressure release	15	13	<LOD–2.00	0.42 (0.11–0.72)	0.03 (0.01–0.13)	2.02 (0.41–9.89)
Other	49	21	<LOD–5.23	0.57 (0.22–0.91)	0.03 (0.01–0.07)	2.35 (1.00–5.49)
**Source**						
Crude oil	219	15	<LOD–22.2	0.66 (0.37–0.94)	0.04 (0.02–0.10)	Reference
Production water	280	31	<LOD–3.45	0.20 (0.14–0.25)	0.01 (0.01–0.04)	0.42 (0.25–0.71)
Wet glycol	65	26	<LOD–3.00	0.30 (0.16–0.44)	0.04 (0.01–0.11)	1.00 (0.40–2.46)
Natural gas	39	26	<LOD–2.00	0.22 (0.07–0.37)	0.01 (0.003–0.03)	0.27 (0.10–0.76)
Condensate	21	10	<LOD–2.10	0.32 (0.07–0.58)	0.07 (0.02–0.31)	1.74 (0.44–6.86)
Mixed benzene sources	53	25	<LOD–0.83	0.07 (0.03–0.11)	0.01 (0.004–0.04)	0.30 (0.12–0.70)
Other benzene sources	86	24	<LOD–3.70	0.31 (0.17–0.46)	0.01 (0.004–0.03)	0.24 (0.11–0.51)
**Season**						
Winter	431	28	<LOD–22.2	0.36 (0.25–0.47)	0.02 (0.01–0.04)	Reference
Summer	332	21	<LOD–11.7	0.33 (0.18–0.48)	0.02 (0.01–0.05)	1.29 (0.77–2.17)
**Indoor/outdoor**						
Outdoor	679	21	<LOD–22.2	0.38 (0.28–0.48)	0.02 (0.01–0.05)	Reference
Indoor	84	43	<LOD–1.51	0.08 (0.03–0.13)	0.01 (0.02– 0.01)	0.22 (0.11–0.45)
**Design of process area**						
Restricted	278	34	<LOD–10.0	0.20 (0.13–0.26)	0.02 (0.01–0.06)	Reference
Partially restricted	385	17	<LOD–22.2	0.45 (0.30–0.61)	0.05 (0.01–0.15)	2.71 (0.48–15.3)
Open	100	24	<LOD–11.5	0.35 (0.08–0.63)	0.07 (0.002–0.03)	0.43 (0.06–1.0)
**Year of production start**						
1979–1989	289	23	<LOD–10.0	0.29 (0.21–0.38)	0.03 (0.01–0.22)	Reference
1990–1999	396	27	<LOD–22.2	0.37 (0.22–0.51)	0.02 (0.01–0.06)	0.60 (0.11–3.40)
2000–2018	78	19	<LOD–4.50	0.44 (0.09–0.79)	0.01 (0.001–0.05)	0.24 (0.03–2.24)
**Sampling method**						
Active	743	25	<LOD–22.2	0.34 (0.25– 0.43)	0.05 (0.01–0.22)	Reference
Passive	20	20	<LOD–4.25	0.57 (−0.05–1.19)	0.02 (0.01–0.04)	2.52 (0.60–10.7)

*N*, number of measurements; LOD, Limit of detection; AM, arithmetic mean; GM, geometric mean; CI, Confidence intervals.

We estimated the overall time trend for task-based benzene exposure using multilevel mixed-effect Tobit regression, which included sampling year as a continuous variable both in the unadjusted model and when adjusted for sampling duration (range 0.5–58 min), task, source, indoor or outdoor work, design of process area, year of production start, sampling method, and sampling duration. To illustrate the time trend overall and for the tasks of sampling and disassembling/assembling (Fig. 2), a linear spline was drawn through the knots in 2007 and 2017, in a scatterplot. For the scatterplots, the measurements below LOD were imputed as *LOD*/√2.

## Results

Overall GM exposure to benzene for the task-based measurements was 0.02 ppm (CI 95%; 0.01–0.04 ppm). The median sampling duration was 7 min (0.5–58 min) (Fig. 1).

Unadjusted analyses of the variables are presented as arithmetic mean (AM), GM, and GM ratios, including corresponding 95% CI in Table 2. The PIG operation was associated with the highest benzene exposure, with a GM of 0.33 ppm (CI 95%; 0.09–1.14 ppm). We divided PIG operation into inserting PIG (0.35 ppm) and receiving PIG (0.37 ppm), to investigate differences between these two work operations, but no statistically significant differences were observed in the unadjusted analyses or when adjusted for sampling duration. Work on the flotation cells was the task with the second highest benzene exposure level, with a GM of 0.16 (0.03–0.82) ppm, followed by disassembling/assembling (GM 0.04 ppm; 0.02–0.10). When conducting laboratory work, the benzene exposure (GM 0.005 ppm; 0.002–0.01) was statistically significant lower compared to conducting sampling (0.01 ppm; 0.01–0.03) with a GM ratio of 0.38. Tasks that included work on process systems containing produced water, natural gas, mixed benzene sources, and other benzene sources were all associated with lower exposure, compared to the handling of crude oil with GM ratios of 0.42, 0.27, 0.30, and 0.24, respectively. The exposure level when working indoors was around 80% lower than when working outdoors. There were no significant differences within the variables’ season, year of production, design of process area, and sampling method.

### Exposure models

Separate exposure models for the *sampling* and *disassembling/assembling* tasks are presented in Table 3.

**Table 3.** Linear mixed-effect models for the *assembling/disassembling* and *sampling* tasks. Random effect: installation. Fixed effects: work operations within assembling/disassembling and sources for assembling/disassembling and sampling.

	Sampling			Disassembling/assembling		
	Model 0	Model 1	*P*-value	Model 0	Model 1	*P*-value
	Random	Fixed		Random	Fixed	
	β (SE)	β (SE)		β (SE)	β (SE)	
**Intercept**	−4.32 (0.33)	−3.58 (0.45)		−3.06 (0.35)	−4.23 (0.62)	
**Variable**						
**Tasks included in disassembling/assembling (work operation)**						
Recertifying or changing valves					Ref	
Changing or cleaning filters					1.62 (0.76)	0.032
Breaking pipes					1.57 (0.72)	0.030
Other assembling/disassembling tasks					0.66 (0.77)	0.391
**Source**						
Crude oil		Ref			Ref	
Produced water		−1.14 (0.43)	0.007		0.16 (0.56)	0.792
Wet glycol		−0.49 (0.60)	0.413		1.05 (0.80)	0.190
Condensate		0.36 (0.76)	0.635			
Natural gas		−1.60 (0.71)	0.024		−1.09 (1.02)	0.287
Mixed sources		0.70 (0.81)	0.411		−1.95 (0.95)	0.041
Other benzene sources		−3.54 (0.63)	0.000		−0.15 (0.62)	0.811
Between-installations variance (_bp_S^2^)	1.56 (0.73)	2.06 (0.90)		1.23 (0.67)	0.63 (0.52)	
Within-installations variance (_wp_S^2^)	7.66 (0.74)	6.53 (0.64)		5.63 (0.71)	5.03 (0.64)	
Total variance (_bp_S^2^ + _wp_S^2^)^*a*^	9.22	8.59		6.86	5.66	
% Variance explained by the fixed effect(s)^*b*^		7			18	

β, regression coefficient; SE, standard error; *P*, probability; ref, reference group.

^*a*^Total variance = bpS^2^ + bwS^2^.

^*b*^% reduction in variance from the random-effect model to the mixed-effect models.

Total variance_(random effects)_ − Total variance_(fixed effects)_ * 100/Total variance_(random effects)_.

For the *sampling* task, the benzene *source* explained only 7% of the total variance in benzene exposure. This exposure model estimates a benzene exposure of 0.03 [^exp^(−3.58)] ppm on sampling crude oil. On collecting samples from the produced water system or from the gas stream, the model estimates benzene exposure at 0.01 ppm. For the *disassembling/assembling* task, the fixed effects *type of work operation* and source explained 18% of the total variance. According to this model, recertifying or changing valves on the crude oil system resulted in exposure of 0.02 ppm. Changing or cleaning filters on the same system was associated with around 5 times higher exposure (0.07 ppm), compared to recertifying or changing valves. The estimated exposure during the breaking of pipes transporting crude oil was 0.07 ppm (Table 3). Among the benzene sources, only the source categorized as wet glycol and mixed had an impact on the exposure relative to crude oil. Changing or cleaning the filter on the wet glycol system was associated with the highest exposure in the model (0.21 ppm).

### Annual changes

**Figure 3.**
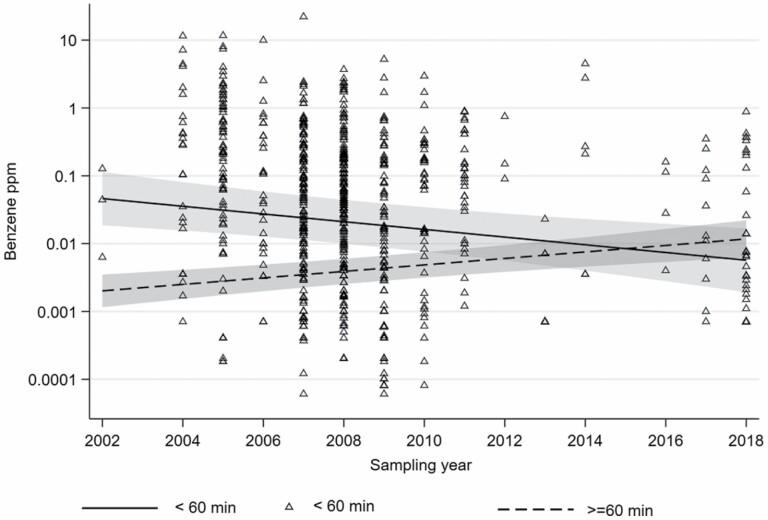
Unadjusted time trend for all task-based measurements with a sampling duration below 60 min (solid line) and as a scatterplot (triangles). In addition, the measurements (equal to or above 60 min sampling duration) presented in Ridderseth *et al.* (2022) are included and presented for comparison (dashed line). Unadjusted splines including knots in the years 2007 and 2017.

During the period from 2002 to 2018, overall task-based benzene exposure declined annually by 10.2% (CI 95%: −17.4 to −2.4%) when adjusted for the variables: task, source, indoor and outdoor, design of process area, year of production start, sampling method, and sampling duration (Supplementary Table S1 is included as supplementary). When analysing the time trend for the tasks of sampling and disassembling/assembling separately, no statistically significant downward trends were seen, −3.9 (−15.0–8.50) and −11.5 (−25.7–5.40), respectively. The time trends for the tasks of sampling and disassembling/assembling were relatively similar, given that the overall CI of −17.4 to −2.43 includes the estimated declines for the two tasks (−11.5% and −3.9%). To illustrate the general time trend, unadjusted splines were drawn through two knots (2007 and 2017 in a scatterplot) (Fig. 3).

## Discussion

The overall task-based GM exposure to benzene was 0.02 ppm, with a median measurement duration of 7 min. PIG operation was the task with the highest benzene exposure, with a GM of 0.33 ppm, followed by work on the flotation cells and disassembling/assembling associated with exposures of 0.16 and 0.04 ppm, respectively. The type of benzene source was a significant determinant of benzene exposure for both the sampling and the disassembling/assembling tasks. In addition, the work operation performed determined benzene exposure for the disassembling/assembling task. Overall, the task-based benzene exposure, comprising ten different tasks, decreased by 10% per year.

The variability of the exposure level was high within the installations for both the sampling and disassembling/assembling tasks (Table 3). The exposure model indicated that the different work operations within disassembling/assembling explained parts of the exposure variability within the installation. Sampling is a more homogeneous task compared to the other tasks (e.g. disassembling/assembling), but the technological design at the different sampling points may vary within the installations. Work habits such as positioning relative to the wind might also affect the exposure when conducting sampling. However, since we did not have information about behavioural issues, the exposure model did not include such factors. For both tasks, the variability in exposure between the installations was lower than the variability within installations, suggesting that any variation in the technology between the installations of relevance for these tasks had less impact on benzene exposure.

When *receiving* the *PIG* from the crude oil pipeline, oil and wax are built up in front of the PIG. Measurement reports described established procedures to reduce wax and sand from the trap before opening and some installations have implemented washing of the PIG before opening the door and removing it. Nevertheless, heavy manual work must be performed in order to remove the PIG. Therefore, we expected higher exposure when receiving the contaminated PIG compared to sending a cleaned PIG. However, there was no statistically significant difference in exposure between receiving and inserting the PIG, which suggests that residual hydrocarbons were still present when opening the door to send the PIG. To the best of our knowledge, there are no other published studies of benzene exposure during PIG operations. However, in 2005 Bråtveit *et al.* (2007) published six measurements with sampling duration of 4–74 min that had been conducted on same task with a GM exposure to benzene of 0.26 (range 0.09–0.67) ppm. Five of these measurements, all with sampling duration below 60 min were included in the present dataset. More measurements were performed in 2006–2017 (21 measurements). There was no statistical difference between the exposure level in 2005 and exposure level in 2006–2017.

*Work on the flotation cells* gave the second highest exposure. A few installations that started production in the 1980s still use this ‘traditional’ flotation technology (DNVGL, 2015), even though new technologies for water treatment have been implemented in this industry. In our dataset, the latest measurements related to working on the flotation cells were from 2011. Most of the measurements were from 2005 and mainly from one installation. For this installation, we have no information about exposure levels after 2005. Inspection of the open flotation cells is only performed when there is a technical issue. Short-term GM exposure of 0.77 ppm (range 0.09–2.33 ppm) was reported by Bråtveit *et al.* (2007) during inspection of the flotation cells in 2005. The measurements are included in the present study, and when analysing exposure for all measurements of work on flotation cells, the exposure level was 0.16 ppm. The measurements performed after 2005 were mainly from collecting water samples from the flotation cells and explain the differences in exposure between the measurements in Bråtveit *et al.* (2007) and when all measurements are included. At present, *hydrocyclones* are a more frequently used rinsing system than ‘traditional’ flotation. This system also needs to be opened occasionally, but the present results indicate that this technology is associated with lower exposure to benzene compared to the flotation cell.

*Disassembling/assembling* was the task with the third-highest exposure level (Table 2). The work operation of *changing or cleaning filter* gave the highest exposure out of four work operations included in the disassembling/assembling task (Table 3). The reason for the high exposure during changing or cleaning of filters might be that in some cases the filters have larger surfaces contaminated with benzene compared to a valve, for example. According to the measurement reports, the equipment was not always drained before they opened and changed the filters, which could indicate that some worst-case measurements are included in the data. Changing or cleaning the filter from the wet glycol system gave higher exposure compared to the same type of work operation on the crude oil system. The benzene content might have been higher in the wet glycol compared to the content in crude oil.

The work operation of the *breaking of pipelines* to transport crude oil was associated with an estimated GM exposure to benzene of 0.07 ppm. To the best of our knowledge, there is no published literature on exposure during the breaking of pipelines offshore. However, at four refineries in the USA, Burns *et al.* (2016) reported that breaking and blinding of undrained pipelines was one of the tasks associated with the highest exposure level (0.06 ppm). However, the exposure level was strongly related to the content of the process stream (Burns *et al.*, 2016). According to procedures on offshore installations, the process system containing crude oil should be drained before opening, but information on any preparation before breaking was lacking in the present study.

The *sampling* task had the highest number of measurements among the tasks investigated. Sampling is conducted daily and several times a day. The exposure level was affected by the benzene source from which the sample was collected. Sampling from the crude oil system gave a higher exposure level compared to sampling from the produced water system, which can probably be explained by the higher benzene content of crude oil compared to produced water. This finding correlates with studies at oil refineries where the benzene exposure level during sampling correlated with the benzene content in the production stream (Verma *et al.*, 2000; Akerstrom *et al.*, 2016). The benzene exposure when sampling from *other sources* was low, as expected since these samples were collected mainly from diesel, which has a low benzene content (<0.02% benzene in diesel, IARC vol 45, 1989).

Working outdoors in the process area was associated with higher benzene exposure compared to working indoors in workshops. Most of the exposure occurs outdoors when opening the process system. The mechanics usually clean the equipment in the process area before bringing the item into the mechanical workshop indoors for further repair or service, which helps to reduce the exposure.

### Annual changes

The annual downward trend of 10% in task-based benzene exposure contrasts with the recently published 7.6% annual upward trend for full-shift benzene exposure among the same group of offshore workers during the same period (2002–2018) (Ridderseth *et al.*, 2022) (Fig. 2). The decline in task-based exposure in the present study indicates that the implementation of control measures and changed work habits might have resulted in reduced exposure for some tasks. However, the implementation of control measures prior to measurements was not sufficiently documented in the measurement reports. On the other hand, the increasing trend for the full-shift measurements might be related to a change in measurement strategy during the last 20 years, from sampling on random days in the first years to a more targeted strategy towards days with expected exposure to benzene (Ridderseth *et al.*, 2022). Studies investigating full-shift exposure in the petroleum industry have commented that a targeted sampling strategy towards days with tasks including benzene exposure is common (Panko *et al.*, 2009; Gaffney *et al.*, 2010; Kreider *et al.*, 2010; Swaen *et al.*, 2010). An alternative explanation for the change in full-shift averages over the years could be that the frequency of benzene-exposed tasks within a workday has increased.

## Strengths and limitations

The strength of this study is the measurements with corresponding contextual information, collected over a period of almost two decades (2002–2018). However, when grouping the measurements into different tasks to build models for estimation of the benzene exposure, the number of measurements in each task category became relatively small for some subgroups. These small numbers hampered the building of models for some tasks, for example PIG operations.

The measurement reports varied in terms of the amount and quality of contextual information, that is from reports that include detailed sampling and process-technical information to reports comprising results solely from exposure measurements. However, the author group comprises industry experts in occupational hygiene that provided additional contextual information, such as which benzene sources were associated with the task-based measurement.

The LOD was not consistently stated in the measurement reports. In most reports, a fixed LOD was given even though the sampling duration varied, while in some reports the LOD varied with sampling duration. Since we did not manage to obtain additional information from the analysing laboratories, we have in our estimations used the LODs as stated in the measurement reports. In this study, measurements lasting for more than 1 h were not included, although in some cases tasks such as work on flotation and recertifying of valves may take more than 1 h. All the personal benzene samplers were placed in the breathing zone, but outside any personal protective equipment (PPE). Hence, the reported benzene levels represent potential, but not necessarily actual, benzene concentrations available for inhalation. At present, there are procedures and guidelines for using PPE when conducting tasks with potentially high exposure, such as during PIG operation and opening of flotation tanks (Norwegian Oil and Gas Association, 2014). Although not consistently described in the measurement reports, several reports stated that respiratory protection was used during the measurement.

## Conclusion

Among 10 typical job tasks performed on offshore installations, PIG operation was associated with the highest exposure to benzene, followed by work on flotation cells and disassembling/assembling. The benzene source was a significant determinant of exposure, with crude oil giving the highest exposure when sampling from the process stream, and wet glycol when disassembling/assembling equipment. Benzene exposure declined over the years. However, since the exposure should be as low as reasonably possible in practice, technical measures should still be considered, especially when opening crude oil process systems for sampling and maintenance such as breaking pipes and changing filters.

## Supplementary Material

wxac067_suppl_Supplementary_Materials

## Data Availability

The data underlying this article are not shared publicly to protect the privacy of organizations that participated in the study, but a summary of raw data is available in the supplemental material.
