# Supplementary material for: Benzene Exposure From Selected Work Tasks on Offshore Petroleum Installations on the Norwegian Continental Shelf, 2002–2018
Source: Ann Work Expo Health. 2022 Oct 21;67(2):228–40. doi: 10.1093/annweh/wxac067 (PMC9923039; doi:10.1093/annweh/wxac067)
Supplement: wxac067_suppl_Supplementary_Materials [file wxac067_suppl_supplementary_materials.docx]

Supplemental materials

**Article title:**

**Benzene exposure from selected work tasks on offshore petroleum installations on the Norwegian continental shelf, 2002-2018**

**Authors:**

Hilde Ridderseth^1^,  Tel: +47 90779086; e-mail: [hilde.ridderseth@uib.no](mailto:hilde.ridderseth@uib.no)

Dagrun Slettebø Daltveit ^1^,

Bjørg Eli Hollund^1^,

Jorunn Kirkeleit^1^,

Hans Kromhout^2^,

Kirsti Krüger^3^,

Kari Aasbø^3^,

Magne Bråtveit^1^

^1^University of Bergen, Bergen, Norway

^2^ Institute for Risk Assessment Sciences, Utrecht University, Utrecht, The Netherlands

^3^Equinor ASA, Norway

**Table 1S.** Annual changes overall and for sampling and disassembling/assembling for the period 2002-2018.

Results are presented unadjusted and adjusted.

|  | **Unadjusted (%)** | **Adjusted (%)** |
| --- | --- | --- |
| **All measurements** | -11.7 (-18.8- to -4.1) | -10.2 (-17.4- to -2.4) |
| **Sampling** | -7.2 (-17.6- 4.6) | -3.9 (-15.0- 8.5) |
| **Disassembling/assembling** | -9.3 (-23.1- 6.9) | -11.5 (-25.7- 5.4) |

**Table 2S.** Linear mixed-effect models for the tasks of *disassembling assembling* and *sampling.*

Random effect: installation and fixed effects: other variables.

|  | **Sampling** | | | **Disassembling/assembling** | | | |  |
| --- | --- | --- | --- | --- | --- | --- | --- | --- |
|  | *Model 0* | *Model 1* |  | | *Model 0* | *Model 1* |  | |
|  | **Random** | **Fixed** |  | | **Random** | **Fixed** |  | |
|  | **β (SE)** | **β (SE)** | **p-value** | | **β (SE)** | **β (SE)** | **p-value** | |
| **Intercept** | -4.32 (0.33) | -3.46 (0.76) |  | | -3.06 (0.35) | -4.47 (0.85) |  | |
| **Variable** |  |  |  | |  |  |  | |
| **Tasks included in assembling/disassembling** |  |  |  | |  |  |  | |
| Recertifying or changing valves |  | NA |  | |  | ref |  | |
| Changing or cleaning filters |  | NA |  | |  | 1.43 (0.79) | 0.071 | |
| Breaking pipes |  | NA |  | |  | 1.52 (0.75) | 0.043 | |
| Other assembling/disassembling tasks |  | NA |  | |  | 0.65 (0.83) | 0.429 | |
| **Source** |  |  |  | |  |  |  | |
| Crude oil |  | ref |  | |  | ref |  | |
| Produced water |  | -1.06 (0.43) | 0.013 | |  | 0.12 (0.61) | 0.846 | |
| Wet glycol |  | -0.51 (0.61) | 0.394 | |  | 1.06 (0.82) | 0.196 | |
| Condensate |  | 0.62 (0.77) | 0.423 | |  |  |  | |
| Natural gas |  | -1.51 (0.71) | 0.033 | |  | -0.89 (1.08) | 0.408 | |
| Mixed sources |  | 1.23 (0.82) | 0.174 | |  | -1.80 (1.00) | 0.070 | |
| Other benzene sources |  | -3.59 (0.63) | 0.000 | |  | 0.26 (0.69) | 0.701 | |
| **Indoor or outdoor** |  |  |  | |  |  |  | |
| Outdoor |  | NA |  | |  | ref |  | |
| Indoor |  | NA |  | |  | -1.09 (1.08) | 0.331 | |
| **Season** |  |  |  | |  |  |  | |
| Winter |  | ref |  | |  | ref |  | |
| Summer |  | -0.21 (0.41) | 0.602 | |  | 0.01 (0.47) | 0.984 | |
| **Design of process area** |  |  |  | |  |  |  | |
| Restricted |  | ref |  | |  | ref |  | |
| Partially restricted |  | 0.48 (0.84) | 0.563 | |  | 0.56 (0.67) | 0.397 | |
| Open |  | -0.26 (0.94) | 0.784 | |  | -0.09 (0.97) | 0.928 | |
| **Sampling method** |  |  |  | |  |  |  | |
| Active |  | ref | 0.173 | |  | ref |  | |
| Passive |  | 1.71 (1.25) |  | |  | 1.01 (1.15) | 0.379 | |
| **Sampling duration** minutes (continuous) |  | -0.02 (0.02) | 0.231 | |  | 0.001 (0.013) | 0.959 | |
| Between-installations variance (_bp_S^2^) | 1.56 (0.73) | 1.96 (0.90) |  | | 1.23 (0.67) | 0.65 (0.60) |  | |
| Within-installations variance (_wp_S^2^) | 7.66 (0.75) | 6.45 (0.63) |  | | 5.63 (0.71) | 4.94 (0.64) |  | |
| Total variance (_bp_S^2^ + _wp_S^2^) *^a^* |  | 8.41 |  | | 6.86 | 5.95 |  | |
| % Variance explained by the fixed effect(s) *^b^* | 9.22 | 9 |  | |  | 19 |  | |

β, regression coefficient; SE, standard error; p, probability; ref, reference group.

*^a^* Total variance = _bp_S^2^ + _bw_S^2^

*^b^* % reduction in variance from random effect model to the mixed-effect models.

Total variance _(random effects)_ – Total variance _(fixed effects)_ * 100/ Total variance _(random effects)._

**Table 1S.** Summary of measurements, measurements below limit of detection, range (minimum-maximum), arithmetic mean, standard deviation, geometric mean, 95% confidence interval and geometric standard deviation are present for each determinant using raw data.

|  | **N** | **N <LOD** | **Range: min-max ppm** |  | **AM ppm** | **SD** |  | **GM ppm** | **95%CI ppm** | **GSD** |
| --- | --- | --- | --- | --- | --- | --- | --- | --- | --- | --- |
| **All measurements** | 763 | 186 | >LOD-22.18 |  | 0.35 | 1.2 |  | 0.03 | 0.02-0.04 | 13.2 |
|  |  |  |  |  |  |  |  |  |  |  |
| **Tasks and work operation** |  |  |  |  |  |  |  |  |  |  |
| Sampling | 355 | 103 | <LOD-11.7 |  | 0.31 | 1.2 |  | 0.02 | 0.05-0.21 | 12.3 |
| Assembling/disassembling | 154 | 12 | <LOD-22-18 |  | 0.50 | 1.9 |  | 0.05 | 0.03-0.08 | 11.9 |
| *Four work operation listed below* |  |  |  |  |  |  |  |  |  |  |
| Recertifying or changing valves | 25 | 6 | <LOD-2.00 |  | 0.18 | 0.5 |  | 0.01 | 0.004-0.03 | 12.3 |
| Changing or cleaning filter | 63 | 3 | <LOD-3.34 |  | 0.43 | 0.7 |  | 0.10 | 0.05-0.17 | 9.4 |
| Breaking pipes | 35 | 2 | <LOD-2.33 |  | 0.45 | 0.8 |  | 0.08 | 0.04-0.16 | 8.7 |
| Other assembling/disassembling tasks | 31 | 1 | <LOD-22.2 |  | 0.94 | 2.7 |  | 0.03 | 0.01-0.07 | 15.2 |
| Laboratory work | 71 | 35 | <LOD-1.51 |  | 0.07 | 0.2 |  | 0.005 | 0.003-0.01 | 12.1 |
| Control of sand trap | 33 | 18 | <LOD-0.40 |  | 0.08 | 0.1 |  | 0.02 | 0.010-0.04 | 9.5 |
| PIG operation | 28 | 0 | 0.03-4.50 |  | 0.60 | 0.9 |  | 0.32 | 0.21-0.49 | 3.1 |
| Skimming | 23 | 3 | <LOD-0.71 |  | 0.13 | 0.20 |  | 0.04 | 0.01-0.10 | 8.3 |
| Work on hydrocyclones | 19 | 4 | <LOD-0.62 |  | 0.13 | 0.2 |  | 0.04 | 0.02-0.10 | 6.2 |
| Work on flotation cells | 15 | 1 | <LOD-2.33 |  | 0.73 | 0.73 |  | 0.27 | 0.09-0.80 | 7.4 |
| Pressure release | 15 | 2 | <LOD-2.00 |  | 0.42 | 0.5 |  | 0.09 | 0.02-0.38 | 12.3 |
| Other | 49 | 8 | <LOD-2.24 |  | 0.56 | 1.2 |  | 0.05 | 0.02-0.10 | 17.4 |
| **Source** |  |  |  |  |  |  |  |  |  |  |
| Crude oil | 219 | 33 | <LOD-22.18 |  | 0.66 | 2.15 |  | 0.05 | 0.04-0.07 | 13.2 |
| Production water | 279 | 88 | <LOD-3.43 |  | 0.20 | 0.5 |  | 0.02 | 0.01-0.03 | 13.6 |
| Wet glycol | 65 | 17 | <LOD-2.96 |  | 0.30 | 0.6 |  | 0.06 | 0.04-0.10 | 7.6 |
| Natural gas | 39 | 10 | <LOD-2.00 |  | 0.22 | 0.5 |  | 0.02 | 0.01-0.05 | 13.2 |
| Condensate | 21 | 2 | <LOD-2.09 |  | 0.32 | 0.6 |  | 0.07 | 0.05-0.19 | 9.2 |
| Mixed benzene sources | 53 | 13 | <LOD-0.83 |  | 0.07 | 0.1 |  | 0.01 | 0.01-0.02 | 7.9 |
| Other benzene sources | 86 | 23 | <LOD-3.70 |  | 0.31 | 0.7 |  | 0.02 | 0.01-0.04 | 16.4 |
| **Season** |  |  |  |  |  |  |  |  |  |  |
| Winter | 430 | 92 | <LOD-11.71 |  | 0.36 | 1.1 |  | 0.03 | 0.02-0.04 | 15.1 |
| Summer | 332 | 94 | <LOD-22.18 |  | 0.33 | 1.4 |  | 0.03 | 0.02-0.04 | 11.0 |
| **Indoor/outdoor** |  |  |  |  |  |  |  |  |  |  |
| Outdoor | 679 | 151 | <LOD-22.18 |  | 0.38 | 1.3 |  | 0.03 | 0.03-0.04 | 12.6 |
| Indoor | 84 | 35 | <LOD-1.51 |  | 0.08 | 0.2 |  | 0.01 | 0.004-0.01 | 12.0 |
| **Design of process area** |  |  |  |  |  |  |  |  |  |  |
| Restricted | 278 | 95 | <LOD-5.24 |  | 0.20 | 0.5 |  | 0.03 | 0.02-0.03 | 10.2 |
| Partially restricted | 384 | 67 | <LOD-22.18 |  | 0.45 | 1.5 |  | 0.04 | 0.03-0.05 | 13.5 |
| Open | 100 | 24 | <LOD-11.53 |  | 0.35 | 1.4 |  | 0.01 | 0.01-0.02 | 19.0 |
| **Year of production start** |  |  |  |  |  |  |  |  |  |  |
| 1979-1989 | 289 | 66 | <LOD-5.24 |  | 0.29 | 0.7 |  | 0.03 | 0.02-0.04 | 12.2 |
| 1990-1999 | 396 | 105 | <LOD-22.18 |  | 0.37 | 1.5 |  | 0.03 | 0.02-0.04 | 12.4 |
| 2000-2018 | 78 | 15 | <LOD-11.53 |  | 0.43 | 1.5 |  | 0.02 | 0.01-0.05 | 22.5 |
| **Sampling method** |  |  |  |  |  |  |  |  |  |  |
| Active | 742 | 182 | <LOD-22.18 |  | 0.34 | 1.2 |  | 0.03 | 0.02-0.03 | 13.2 |
| Passive | 20 | 4 | <LOD-4.43 |  | 0.57 | 1.3 |  | 0.05 | 0.02-0.16 | 12.3 |

N; number of measurements, LOD; limit of detection, AM; arithmetic mean, SD; standard deviation, GM; geometric mean, CI; confidence interval, GSD, geometric standard deviation

To calculate AM, SD, GM, 95% CI and GSD, measurements below LOD were divided with 2–√*2*
